# Tocilizumab Effect on Lipid Profile in Correlation to Cardiovascular Events: A Retrospective Cohort Study

**DOI:** 10.1155/2021/5535486

**Published:** 2021-08-12

**Authors:** Toka Alsulaim, Noor Alhassan, Hala Khalil, Abdullah Almutlaq

**Affiliations:** ^1^Rheumatology Division, Department of Medicine, King Faisal Specialist Hospital and Research Centre, Riyadh, Saudi Arabia; ^2^Department of Biostatistics, Epidemiology and Scientific Computing, King Faisal Specialist Hospital and Research Centre, Riyadh, Saudi Arabia

## Abstract

**Objective:**

To study the effect of tocilizumab initiation on the lipid profile, in correlation to a composite of any cardiovascular events.

**Methods:**

A retrospective cohort study, using data from the King Faisal Specialist Hospital & Research Centre database, from January 2014 to December 2019. Patients with rheumatoid arthritis or juvenile idiopathic arthritis who were ≥18 years old, initiated either on tocilizumab or other biologic treatment (anti-TNFs or Rituximab), were included, with a follow-up interval duration at a minimum of 6–12 months up to 3-5 years. Any patient with established cardiovascular disease or aged <18 were excluded.

**Results:**

Only one cardiovascular mortality was reported in the tocilizumab group. Fifty percent of patients reached high cholesterol levels ≥ 5.2 mmol/L and LDL ≥ 3.37 mmol/L in the tocilizumab group at 36 months in a shorter time period compared to controls (60 months), *P* 0.001. There were no significant differences between groups for statin use (27% vs. 28%) However, there was a significantly higher mean dose of atorvastatin in the tocilizumab group compared to controls (20.6 mg vs. 16.6 mg, *P* 0.03).

**Conclusion:**

There was a lack of evidence of increased cardiovascular risk in correlation to hyperlipidemia secondary to tocilizumab treatment.

## 1. Introduction

The interleukin-6 (IL-6) inhibitors, one of which is tocilizumab, a monoclonal autoantibody, has been studied and approved for rheumatoid arthritis treatment back in 2010 along with other autoimmune diseases including systemic-onset juvenile idiopathic arthritis, Castleman's disease, multiple myeloma, systemic lupus erythematosus, and, lately, giant cell arteritis in 2017. It has been given either subcutaneously once weekly or every four weeks intravenously in a dose of 4-8 mg/kg and found to reduce disease activity, induce remission, and reverse joint damage in early stages of rheumatoid arthritis [1]. The effect of tocilizumab on the lipid profile is not well understood yet, as it has been observed in the literature that hyperlipidemia is the most common side effect of tocilizumab; however, the mechanism underlying this is unclear [2, 3].

Theoretically, IL-6 is a cytokine that plays an important role in autoimmune and inflammatory regulation, and its level is directly correlated to insulin resistant and free fatty acid by increasing the adipocyte lipolysis of triglycerides. Given that tocilizumab is an IL-6 inhibitor which blocks the IL-6 receptor, it was found that IL-6 levels appear to be increased after treatment, which suggests that the mechanism underlying dyslipidemia is due to the direct effect of IL-6 [4].

Its safety profile and efficacy were studied, where it has been noted that the adverse effects are similar to that noted in the methotrexate control group (79.9% tocilizumab vs. 77.5% methotrexate; *P* = 0.484) except that for higher total cholesterol and LDL in the tocilizumab group but with no reported increase in cardiovascular morbidities or mortalities, and its clinical significance is unclear till now which requires further long-term follow-up [5]. An open-labelled, phase 4 study conducted throughout 5 countries (Bahrain, Iran, Kuwait, Qatar, and UAE) between January 13, 2010, and June 20, 2011, did show a 10.5% increase in blood cholesterol among the 95 participants [6].

Rheumatoid arthritis and almost all other autoimmune diseases are associated with the risk of cardiovascular disease through systemic inflammation by direct and indirect effects [7], and the relation between disease activity and treatments, mainly IL-6 tocilizumab and cardiovascular risk, has never been studied in the Middle East and especially in the Kingdom of Saudi Arabia.

## 2. Materials and Methods

### 2.1. Data Sources

We conducted a retrospective cohort study using data from King Faisal Specialist Hospital (KFSH) Integrated Clinical Information System (ICIS) Database, which is a local system which contains longitudinal medical and pharmaceutical data from several different managed care plans (emergency, inpatient, and outpatient care services), by collecting data through all adult rheumatology patients followed in KFSH, Riyadh, from January 2014 to December 2019.

The study was carried out in accordance with the ethical principles of the Helsinki Declaration and King Faisal Specialist Hospital policies and guidelines for clinical research.

Patient privacy was protected by keeping the data sealed with a password known only by the investigators. The Institutional Review Board (IRB) of the King Faisal Specialist Hospital & Research Centre approved the study protocol and privacy precautions.

### 2.2. Study Design

We identified adult patients aged ≥18 years old with a diagnosis of rheumatoid arthritis or juvenile idiopathic arthritis who have been initiated either on tocilizumab (whether a bDMARDs naïve or with previous use of bDMARDs) or other biologic treatment (anti-TNFs or Rituximab) who at least have baseline lab results that include lipid profile prior starting the biologic treatment with follow-up duration interval after starting therapy in the clinic or day medical unit for a minimum of 6–12 months up to 3-5 years.

Patients were excluded if there was any history of established cardiovascular disease including the history of unstable angina, nonfatal MI, and nonfatal stroke. We have also excluded patients aged less than 18 years old at the time of initiation of the biologic treatment.

### 2.3. Outcome Assessment

The primary outcome was the effect of therapy initiation on the lipid profile, having hyperlipidemia as defined by the National Cholesterol Education Program (NCEP) USA (Table 1) which is used in King Faisal Specialist Hospital & Research Centre in correlation to a composite of any cardiovascular event—if any—after treatment initiation including nonfatal MI, unstable angina, nonfatal stroke, and cardiovascular mortality.

The secondary outcome includes assessing the effect of dose escalation of tocilizumab on the lipid profile and cardiovascular events, in addition to having a view on the percentage of patients using lipid-lowering agents in both groups and the risk of developing cardiovascular event.

For covariant assessment, we have measured baseline variables potentially related to cardiovascular risk including (hypertension, diabetes mellitus, dyslipidemia, body mass index, and smoking history), in addition to other prescribed treatment including cDMARDs and lipid-lowering agents. We have also assessed demographics, time of diagnosis, and other comorbidities.

### 2.4. Statistical Analysis

The data were examined for normality by visual inspection of histograms and the Kolmogorov-Smirnoff test. For comparison of the study groups at baseline, we used *T*-tests or nonparametric Mann–Whitney test for continuous variables and a chi-square (*χ*^2^) analysis for categorical variables. Bivariate comparisons of change in lipoprotein levels at 3 and 6 months were conducted using the paired sample *T*-test or the related sample Wilcoxon signed-rank test as appropriate. Due to the nature of our study design as a retrospective chart review study, we have a fixed sample size, so a poststudy power analysis was not conducted as it is of little value.

We used the Kaplan–Meier (KM) curve to describe the changes in lipoprotein levels over the observation period. For comparing KM curves, we used the log-rank test (Mantel–Haenszel) to assess differences between the two groups. Also, we used mixed-effect regression models to assess the difference in the change of lipid levels between the two groups over the follow-up period. A *P* value < 0.05 was considered statistically significant in the univariate or multivariate model. The relationship between tocilizumab dose and levels of cholesterol, triglycerides, LDL, and HDL was assessed using partial correlations, adjusted for other predictors.

## 3. Results

**Figure 1:**
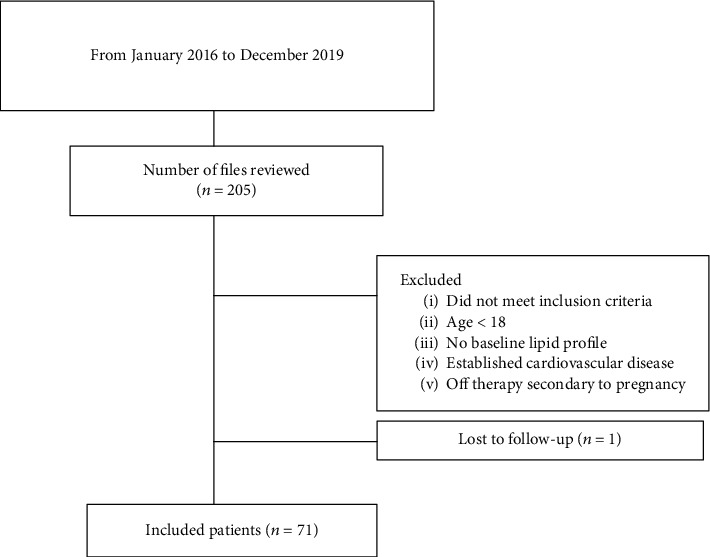
Study design: patient selection.

A total of 71 patients fulfilling the inclusion criteria were identified and enrolled in the tocilizumab group (Figure 1) with 72 patients in the control group receiving biological DMARDs (anti-TNFs or Rituximab) who never initiated tocilizumab at any time.

Baseline demographics were similar among groups, including gender, which noted more women than men in both (83% in the tocilizumab group, 88% in the control group), and other potential cardiovascular risk factors, for example, smoking history and BMI. However, the mean age was higher in the controls than the treatment group in a significant value (50 ± 11.7 and 44 ± 12.8, *P* 0.001) (Table 2) and the mean duration of disease was (11.57 ± 7.93 and 7.03 ± 6.49) in tocilizumab and control groups respectively. Concomitant chronic illnesses were reported with no significant statistical differences (Table 3).

At baseline, the median lipid profile was statistically not different between the groups (Table 4). There were no cardiovascular events (stable angina, unstable angina, nonfatal myocardial infarction, or stroke) noted or reported in any of the groups. Only one cardiovascular mortality was reported in the tocilizumab group.

A Wilcoxon signed-rank test conducted within group change in lipoprotein levels at different time points through the retrospective collected data did show that the median lipoprotein level ranks at baseline were significantly higher than the median level ranks at the indicated time point (Table 5). In the tocilizumab group, for example, HDL levels increased within 3 months significantly with a *P* value of 0.04, where at the same time, there were no changes in the lipoprotein levels in the control group.

In 6-month, 12-month, and 18-month follow-ups, there was a significant increase in both total cholesterol (*P* < 0.001, 0.03, <0.001) and LDL-C (*P* 0.01, <0.001, <0.001), in the control group analysis.

Fifty percent of patients reached a high cholesterol level ≥ 5.2 and LDL ≥ 3.37 in the tocilizumab group at 36 months compared to the control group where half of the patients reached the median in a longer time period of 60 months (log-rank test, *χ*^2^ (1) = (10.71 and 9.10), respectively, *P* = 0.001). On the other hand, there was no differences between groups in triglyceride levels (log-rank test, *χ*2 (1) = 3.84, *P* = 0.05).

**Figure 2:**
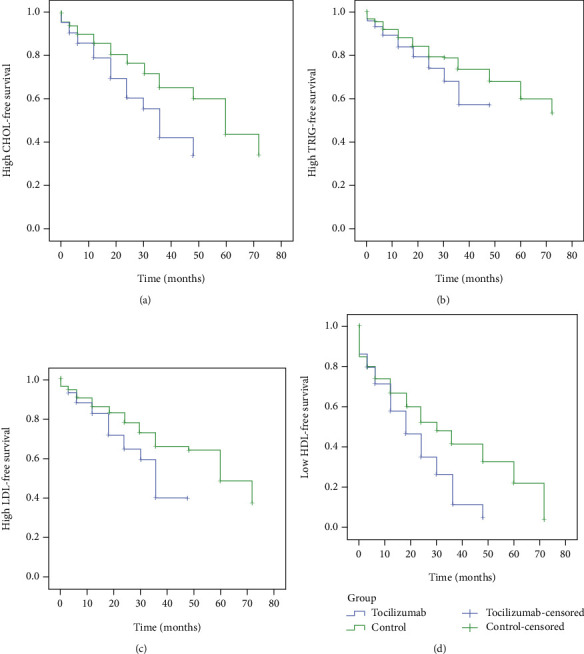
Kaplan–Meier estimate of the time-to-variable lipid profile elevation. (a) The median survival time (high CHOL-free time) for the treatment group was 36 months and for the control group 60 months. The median survival time was significantly longer in the control than in the treatment group (log-rank test, *χ*^2^ (1) = 10.71, *P* = 0.001). (b) The median survival time (high TRIG-free time) for the treatment group was 36 months and for the control group 55 months. The median survival time was not significantly different between the control and the treatment group (log-rank test, *χ*^2^ (1) = 3.84, *P* = 0.05). (c) The median survival time (high LDL-free time) for the treatment group was 36 months and for the control group 60 months. The median survival time was significantly longer in the control than in the treatment group (log-rank test, *χ*^2^ (1) = 9.10, *P* < 0.001). (d) The median survival time (high HDL-free time) for the treatment group was 18 months and for the control group 30 months. The median survival time was significantly longer in the control than in the treatment group (log-rank test, *χ*^2^ (1) = 20, *P* < 0.0001).

The median survival time for high HDL-free time in the treatment group was 18 months, and for the control group, 30 months, followed up for 6 years, which was a significantly longer time in the control than in the treatment group (log-rank test, *χ*^2^ (1) = 20, *P* < 0.0001) (Figure 2).

By further analyzing the data, taking in consideration the BMI, there was no remarkable finding in the lipid profile in both groups only after 6 months, where significant changes were found among patients with BMI lower than 30 in the tocilizumab group in the LDL level at 6, 18, and 24 months in comparison to the control group.

Patients were using statin (atorvastatin being the most used medication) as a lipid-lowering agent at any time point of the study with no significant differences between both studied groups (27%-28%). However, a significantly higher mean dose of atorvastatin was used in the tocilizumab group compared to the controls (20.6 mg vs. 16.6 mg, *P* 0.03). A correlation between cholesterol and lipoprotein levels with tocilizumab dose, at different time points adjusted to multiple variables such as baseline lipid profile levels and age at diagnoses, was conducted which notes a statistical increase in triglyceride levels by the 18-month treatment duration (*P* < 0.05) (Table 6).

## 4. Discussion

Tocilizumab has been studied thoroughly as a well-known cause of subsequent hyperlipidemia [8–11]; however, cardiovascular safety was not fully linked to this lipid profile risk; multiple randomized control trials and descriptive studies failed to link any risk of a cardiovascular event to the use of tocilizumab in comparison to other biologic therapies [10–14].

In this cohort, our results were comparable to other studies in terms of lack of evidence of increased cardiovascular risk in correlation to hyperlipidemia secondary to tocilizumab treatment. Only one cardiovascular mortality was encountered in the tocilizumab group, reported as in-hospital mortality, secondary to an acute coronary syndrome, after a complicated hospital course and multiple comorbidities including COPD, diabetes, and hypertension in an advanced aged patient. However, it is important to note that tocilizumab was held for a few months prior to her death due to recurrent admissions for multiple infections. In addition to that, her lipid profile was consistently normal with no reported escalation of the dose of tocilizumab, so linking such an individual event would not reflect the direct effect of tocilizumab on cardiovascular safety given the normal lipid profile in her case.

Progressive significant hyperlipidemia was reported in both groups as expected; however, lipoprotein levels were significantly higher in the control group in comparison to the tocilizumab group at different time points, unlike what was reported in a study by Gabay et al., through a post hoc analysis comparing the lipid profile between tocilizumab and adalimumab, as a significant increase was found in LDL and HDL with tocilizumab than adalimumab at 24 weeks [15]. However, in the subgroup analysis, when taking into account the BMI of patients, it was noted that significant high LDL difference was encountered—interestingly—in the lower BMI patients in the tocilizumab group at 6, 18, and 24 months in comparison to the control group.

The significant difference that was observed between the two groups in our study could be secondary to unmeasured factors, for example, disease activity, which was not assessed; in addition to that, an older age group was noted in the controls that might have resulted in some confounding. It is worth mentioning that overall lipid-lowering agent (atorvastatin) use percentage at any point during the study among the patients was equal in both groups; however, the mean dose in the group that was treated with tocilizumab was higher than the mean dose that was used in the control group (20.6 mg vs. 16.9 mg; *P* 0.03) which might confound the lipid levels that were observed in the tocilizumab group, keeping in mind that no direct correlation was tested between the statin use/dose and lipid levels in our study.

Regarding the hyperlipidemia pattern, it was interesting to note that more patients in the tocilizumab group were found to get dyslipidemia mainly (high total cholesterol and LDL levels) in shorter time duration in comparison to the control group with a mean duration of 36 and 60 months, respectively (*P* 0.001); no similar finding was described previously in the literature to compare such a result, but some studies have described progressive hyperlipidemia with tocilizumab in a short time as early as 12 weeks which persists until week 24, which almost returned to the baseline at 52-104 weeks [16–18]; no direct comparison with other biologics was described.

The strength of our study is mainly captured by being a cohort study with two groups comparing tocilizumab with other biologic therapies which provides further significance to the tested outcome; in addition to that, our study took into account many potential covariates that might confound any association, mainly by excluding any patient with previous cardiovascular event, in addition to nearly matched baseline characteristics between both groups including some of the cardiovascular risk factors, for example, smoking, obesity, and other comorbidities like hypertension and diabetes. It is the first study that can be found in the Middle East, specifically Saudi Arabia, as there were no previous local studies that were conducted to assess the studied topic.

On the other hand, limitations of the study can be recognized by the short duration of follow-up for the patients who have been started on the medication, given that a cardiovascular event would be expected to be a long-term sequel of hyperlipidemia; however, the recent introduction of tocilizumab in the facility was no longer than six years with a limited number of patients and follow-up lipid profile. We can add that our center, by being a large tertiary referral center, would limit some presentation or reporting cardiovascular event or mortality, as most of our patients are from peripheral areas and would present in their local hospital for any such event, limiting our data especially for cardiovascular mortality.

## 5. Conclusion

Lack of Evidence of increased cardiovascular risk in correlation to progressive reported hyperlipidemia secondary to tocilizumab treatment. This was parallel to international randomized control studies.

## Figures and Tables

**Table 1:** The National Cholesterol Education Program (mmol/L).

Triglyceride	Low density lipoprotein (LDL)	High density lipoprotein (HDL)	Cholesterol
Normal: <1.7	Optimal: <2.56	Low HDL: <1.04	Desirable: <5.2
Borderline high: 1.7–2.25	Low risk: 2.59–3.34	Normal: 1.04–1.55	Borderline high: 5.2–6.2
High: 2.26–5.64	Borderline high: 3.37–4.12	Desirable: >1.55	High: >6.2
Very high: >5.64	High: 4.14–4.90		
	Very high: >4.9		

**Table 2:** Clinical characteristics at baseline: demographics.

	Tocilizumab (*N* = 71)	Control (*N* = 72)	Total (*N* = 143)	*P* value
Gender	*N* (%)			
Male	12 (17)	9 (12)	21 (15)	0.46
Female	59 (83)	63 (88)	122 (85)	
Age (years)	44 ± 12.8	50.7 ± 11.7	47.4 ± 12.7	0.001^∗^
BMI (kg/m^2^)	30.4 ± 6.9	30.6 ± 5.3	30.5 ± 6	0.78
Smoker				
Yes	3 (7)	0 (0)	3 (4)	0.21
No	39 (93)	41 (100)	80 (96)	

^∗^Significant *P* value < 0.05.

**Table 3:** Clinical characteristics at baseline: concomitant illness.

	Tocilizumab (*N* = 71)	Control (*N* = 72)	Total (*N* = 143)	*P* value
Diabetes mellitus	13 (18)	17 (24)	30 (21)	0.44
Hypertension	15 (21)	15 (21)	30 (21)	0.97
Hyperlipidemia	7 (10)	6 (8)	13 (9)	0.75
Liver diseases	0 (0)	2 (3)	2 (1)	0.16
CKD	1 (1)	1 (1)	2 (1)	0.99
Statin use	19 (27)	20 (28)	39 (27)	0.89

**Table 4:** Clinical characteristics at baseline: lipid profile.

	Tocilizumab	Control	Total	*P* value
	Median (min, max)			
HDL-C (mmol/L)	1.5 (0.6, 2.45)	1.7 (0.8, 5.0)	1.5 (0.6, 5.2)	0.93
TRIG (mmol/L)	1.0 (0.5, 5.9)	1.0 (0.4, 5.0)	1.0 (0.4, 5.9)	0.58
	Mean ± SD			
LDL-C (mmol/L)	2.7 ± 0.7	2.7 ± 0.7	2.7 ± 0.7	0.84
Total CHOL (mmol/L)	4.5 ± 0.8	4.5 ± 0.7	4.5 ± 0.8	0.86

**Table 5:** Lipoprotein level changes at an indicated time point.

	Tocilizumab		Control	
Within group change in lipoprotein levels at 3 months				
	Mean differences (SD)	*P*	Mean differences (SD)	*P*
Total CHOL	0.21 (0.65)	0.09	0.07 (0.5)	0.57
LDL-C (mmol/L)	0.12 (0.64)	0.29	0.21 (0.74)	0.27
	Mean differences; *Z*	*P*	Mean differences; Z	*P*
TRIG^∗^ (mmol/L)	0.05; -1.41	0.16	0.10; -0.48	0.63
HDL-C^∗^ (mmol/L)	0.13; -2.01	0.04^∗∗^	0.03; -0.99	0.32
Within group change in lipoprotein levels at 6 months				
	Mean differences (SD)	*P*	Mean differences (SD)	*P*
Total CHOL	0.46 (1.13)	0.04^∗∗^	0.35 (0.64)	<0.001^∗∗^
LDL-C (mmol/L)	0.39 (1.11)	0.07	0.30 (0.58)	0.01
	Mean differences; *Z*	*P*	Mean differences; *Z*	*P*
TRIG^∗^ (mmol/L)	0.18; -1.35	0.18	0.14; -1.41	0.16
HDL-C^∗^ (mmol/L)	0.15; -2.40	0.02^∗∗^	-0.08; -1.49	0.14
Within group change in lipoprotein levels at 12 months				
	Mean differences (SD)	*P*	Mean differences (SD)	*P*
Total CHOL	0.14 (0.81)	0.27	0.35 (0.76)	0.03^∗∗^
LDL-C (mmol/L)	0.11 (0.83)	0.37	0.39 (0.63)	<0.001^∗∗^
	Mean differences; *Z*	*P*	Mean differences; *Z*	*P*
TRIG^∗^ (mmol/L)	0.10; -0.87	0.39	0.12; -1.41	0.16
HDL-C^∗^ (mmol/L)	0.12; -1.92	0.06	-0.19; -0.27	0.79
Within group change in lipoprotein levels at 18 months				
	Mean differences (SD)	*P*	Mean differences (SD)	*P*
Total CHOL	0.21 (0.67)	0.12	0.58 (0.80)	<0.001^∗∗^
LDL-C (mmol/L)	0.39 (0.53)	<0.001^∗∗^	0.48 (0.72)	<0.001^∗∗^
	Mean differences; *Z*	*P*	Mean differences; *Z*	*P*
TRIG^∗^ (mmol/L)	-0.10; -0.49	0.62	0.09; -1.26	0.21
HDL-C^∗^ (mmol/L)	-0.01; 0.00	1.00	0.01; -1.03	0.30
Within group change in lipoprotein levels at 24 months				
	Mean differences (SD)	*P*	Mean differences (SD)	*P*
Total CHOL	0.38 (0.83)	0.047^∗∗^	0.36 (0.69)	0.01^∗∗^
LDL-C (mmol/L)	0.33 (0.71)	0.045^∗∗^	0.23 (0.79)	0.15
	Mean differences; *Z*	*P*	Mean differences; *Z*	*P*
TRIG^∗^ (mmol/L)	-0.05; -0.10	0.92	0.18; -0.85	0.40
HDL-C^∗^ (mmol/L)	0.14; -1.86	0.06	0.01; -0.72	0.47

^∗^Wilcoxon signed-rank test conducted; ^∗∗^Significant *P* value <0.05.

**Table 6:** Correlation between cholesterol and lipoprotein levels with tocilizumab dose, at different time points.

	3 months (*n* = 17)			
Tocilizumab dose	CHOL	TRIG	LDL	HDL
Correlation coefficient^‡^ (*r*)	0.002	-0.28	-0.19	0.34
	6 months (*n* = 30)			
Tocilizumab dose	CHOL	TRIG	LDL	HDL
Correlation coefficient^‡^ (*r*)	-0.05	0.08	-0.11	0.19
	12 months (*n* = 24)			
Tocilizumab dose	CHOL	TRIG	LDL	HDL
Correlation coefficient^‡^ (*r*)	0.34	0.41	0.32	-0.04
	18 months (*n* = 14)			
Tocilizumab dose	CHOL	TRIG	LDL	HDL
Correlation coefficient^‡^ (*r*)	-0.03	0.78^∗^	0.15	-0.27
	24 months (*n* = 27)			
Tocilizumab dose	CHOL	TRIG	LDL	HDL
Correlation coefficient^‡^ (*r*)	-0.25	0.01	-0.36	0.14
	30 months (*n* = 5)			
Tocilizumab dose	CHOL	TRIG	LDL	HDL
Correlation coefficient^‡^ (*r*)	-0.99	-0.56	-0.93	0.24
	36 months (*n* = 12)			
Tocilizumab dose	CHOL	TRIG	LDL	HDL
Correlation coefficient^‡^ (*r*)	-0.30	-0.39	-0.33	0.28

^‡^Controlled for age at diagnosis and baseline level of cholesterol or lipoproteins. ^∗^Statistical significance at *P* < 0.05. ^∗∗^Statistical significance at *P* < 0.01.

## Data Availability

The Institutional Review Board (IRB) of King Faisal Specialist Hospital & Research Centre approved the study protocol and privacy precautions. Data are available on request from the authors who meet the criteria for access to confidential data.
